# Supplementary material for: Interstellar ices as carriers of supernova material to the early solar system
Source: Nat Commun. 2025 Nov 27;16:10657. doi: 10.1038/s41467-025-65672-5 (PMC12660829; doi:10.1038/s41467-025-65672-5)
Supplement: Supplementary file 1 — Supplementary Information [file 41467_2025_65672_MOESM1_ESM.pdf]

Supplementary Information for

Interstellar Ices as Carriers of Supernova Material to the Early Solar System

Martin Bizzarro[1,2]*, Martin Schiller[1], Jesper Holst[1], Laura Bouvier[1], Mirek Groen[1], Frédéric Moynier[2], Elishevah van Kooten[1], Maria Schönbächler[3], Troels Haugbølle[4], Darach Watson[4], Anders Johansen[1,5], James Connelly[1], Emil Bizzarro[1]

1–Center for Star and Planet Formation, Globe Institute, University of Copenhagen, Copenhagen, Denmark. 2–Institut de Physique du Globe de Paris, Université de Paris Cité, Paris, France. 3–Institute for Geochemistry and Petrology, ETH Zürich, Zürich, Switzerland. 4–Center for Star and Planet Formation, Niels Bohr Institute, University of Copenhagen, Copenhagen, Denmark. 5–Lund Observatory, Department of Astronomy and Theoretical Physics, Lund University, Lund, Sweden

*Corresponding author. Email: bizzarro@sund.ku.dk

This PDF file includes:

Supplementary Text

Figs. S1 to S4

Tables S1

Data S1

Supplementary Text

Mass balance calculations to estimate the Zr content of ices

A key observation of our paper is that all Solar System objects lie on a linear mixing relationship between the residue and ice end member compositions. This suggests that the variability in the Zr isotope composition of bodies and solids may be a function of the proportion of ice accreted. To infer the relative proportion of ices accreted by the various bodies, knowledge of the concentration of Zr present in the ices is required. This quantity can be derived by mass balance calculations. Additionally, knowledge of the Zr concentration of ices can also be used to evaluate whether the Zr present in the L3 leachate is predominantly from the ices, or alternatively, represent a mixture between the silicate and ice components. For example, if a non-negligeable fraction of the Zr recovered in the L3 leachate is derived from the silicate component, this would lead to overestimating the concentration of Zr in the ices. While this does not impact the main results and interpretation of our paper, it allows us to explore the extent to which most anomalous Zr isotope compositions defined by the Tagish Lake, Maribo and Murchison L3 fractions represent a diluted signal of the true ice endmember composition. This information can also be used to evaluate the dilution factor of the Zr isotopic signal of the true ice endmember composition relative to presolar grains and supernovae models.

We define the following quantities:

$[Zr]_T$ = zirconium concentration in bulk parent body

$[Zr]_I$ = zirconium concentration in ice

$[Zr]_R$ = zirconium concentration in silicate

F_I = mass fraction of ice in chondrite

Thus,

$$[Zr]_T = F_I[Zr]_I + (1 - F_I)[Zr]_R \quad (1)$$

$$[Zr]_T = F_I[Zr]_I + [Zr]_R - F_I[Zr]_R \quad (2)$$

$$[Zr]_T - [Zr]_R = F_I[Zr]_I - F_I[Zr]_R \quad (3)$$

$$F_I = \frac{[Zr]_T - [Zr]_R}{[Zr]_I - [Zr]_R} \tag{4}$$

We can use the Zr concentration data from the leaching experiments to define the relative proportion of Zr in the ice and residue. We focus on the CM chondrites, namely Maribo and Murchison, for which the highest amount of Zr was recovered from the L3 fractions. Because only negligeable amounts of Zr are present in the L1 and L2 fractions, we use the L3 to constrain the amount of Zr present in the ice relative to the residue. Maribo and Murchison defined similar residue to L3 ratios of 5.3 and 4, respectively. However, given that Maribo is the most pristine of the two CMs, we retain the value of 5.3 in our calculations.

$$\frac{1}{5.3} = \frac{F_I[Zr]_I}{(1 - F_I)[Zr]_R} \tag{5}$$

$$(1 - F_I)[Zr]_R = \ 5.3F_I[Zr]_I \tag{6}$$

$$[Zr]_R = \frac{5.3F_I[Zr]_I}{(1 - F_I)} \tag{7}$$

Replacing $[Zr]_R$ in equation (4) gives:

$$F_I = \frac{[Zr]_T - \frac{5.3F_I[Zr]_I}{(1 - F_I)}}{[Zr]_I\left(1 - \frac{5.3F_I}{(1 - F_I)}\right)} \tag{8}$$

$$F_I[Zr]_I\left(1 - \frac{5.3F_I}{(1 - F_I)}\right) = [Zr]_T - \frac{5.3F_I[Zr]_I}{(1 - F_I)} \tag{9}$$

$$F_I[Zr]_I\left(1 - \frac{5.3F_I}{(1 - F_I)}\right) = [Zr]_T - \frac{5.3F_I[Zr]_I}{(1 - F_I)} \tag{10}$$

$$[Zr]_T = [Zr]_I\left(F_I - \frac{5.3F_I^2}{(1 - F_I)} + \frac{5.3F_I}{(1 - F_I)}\right) \tag{11}$$

$$[Zr]_I = \frac{[Zr]_T}{\left(F_I - \frac{5.3F_I^2}{(1-F_I)} + \frac{5.3F_I}{(1-F_I)}\right)} \qquad (12)$$

The amount of ice accreted by CM chondrites, defined here as F_I, has been estimated by various studies and approaches and ranges from 10 to 40% by mass[1–3]. We use a value of 25% (ice-to-rock ratio of 0.25), which represents the average value for the range of estimates. Using a value of 0.25 for F_I and the solar Zr concentration of 3.6 ppm[4] for the $[Zr]_T$ parameter returns a Zr concentration in the ice ($[Zr]_I$) of 2.3 ppm. This estimate, however, is based on the assumption that the totality of the Zr recovered from the L3 fraction is derived from the ices. This may not be realistic because the Zr recovered from the L3 fraction is essentially derived from the phyllosilicates, which are formed by the interaction of water and preexisting silicate minerals, including amorphous silicates as well as olivine and pyroxene[5]. As such, a non negligeable amount of Zr in the L3 fraction must have been originally hosted by these silicate phases prior to the onset of aqueous alteration such that the inferred Zr ice concentration of 2.3 ppm represents an upper limit. As noted in the main text, the Zr isotopic composition of the most anomalous L3 fractions (i.e. Tagish Lake, Maribo and Murchison) represents a dilute composition of a supernova component that can be best approximated by that of presolar graphite grains. The average μ^{96}Zr value of the presolar graphite grains is nearly three orders of magnitude greater than that of the L3 fractions. From theoretical considerations[6,7], the Zr supernova component present in the ices should be diluted by one to two orders of magnitude based on the proportion of processed fresh supernova dust relative to the ambient ISM dust reservoir, which is at least a factor 10 less than deduced from the leachate data. Thus, we conclude that $[Zr]_R/[Zr]_I$ value of 5.3 used in the mass balance calculation is underestimated by at least a factor of 10. Recalculating the Zr concentration in the ices ($[Zr]_I$) using a $[Zr]_R/[Zr]_I$ ratio of 53 as opposed to 5.3 returns a $[Zr]_I$ value of 0.27 ppm, which we infer is a strict maximum for the Zr concentration of the ices. We emphasize that this level of uncertainty regarding the Zr concentration of the ices and, by extension, the Zr isotopic composition of the pure ice member does not impact the main conclusions presented in the paper.

<u>Comparison with recent literature data</u>

Several papers have recently reported Zr isotope data for bulk meteorites[8–12], refractory inclusions[13] and step leaching experiments[10,11]. In particular, three reports from one laboratory[8,10,12] include all the chondrite and achondrite groups analyzed in our study, which provides a basis for comparison. We compare our μ^{96}Zr data to that summarized by Render *et al.*[10] for the same meteorite groups in Fig. S3a. Although there is a very good agreement for achondrites (Mars, HED and APB) as well as chondrites with low matrix abundances such as EC and OC, some discrepancy is apparent for the three carbonaceous chondrites (CO group,

Murchison and Orgueil) analyzed by each laboratory. For example, the μ^{96}Zr data for Orgueil and Murchison reported in Render *et al.*[10] is more anomalous than our values. Our digestion procedure for bulk meteorites involved a high pressure and temperature digestion step in Parr™ bombs to ensure successful digestion of refractory components such as highly anomalous SiC. In contrast, the samples of Orgueil and Murchison analyzed by Render *et al.*[10] were hot plate digestions, which does not ensure full digestion of refractory components[9]. We note that the μ^{96}Zr composition of Orgueil has also been analyzed by Akram *et al.*[9], using a bomb digestion approach similar to that used in our study. We show in Fig. S3b our three sample digestion of Orgueil together with that of Akram *et al.*[9] and Render *et al.*[10]. Although of lower resolution, the Orgueil μ^{96}Zr data reported by Akram *et al.*[9] is fully consistent with our value but not with that of Render *et al.*[10]. We speculate that the Orgueil and Murchison data in Render *et al.*[10] and possibly other data they report for carbonaceous chondrites are affected by analytical artifacts associated with the lack of full sample dissolution. Our μ^{96}Zr data for the CO chondrite SAH 99544 is more anomalous outside of analytical uncertainty than reported by the CO chondrite Kainsaz by Render *et al.*[10]. This discrepancy is difficult to ascribe to analytical artifacts related with the lack of full sample dissolution as our procedure involves bomb digestion. A possibility is that heterogeneity exists amongst the CO group, which is consistent with data reported by Akram *et al.* [9] although these have large uncertainties. We note that Elfers *et al.*[11], which utilize a bomb digestion approach similar to that used in our study, report μ^{96}Zr data for Kainsaz that agrees with Render *et al.*[10], thereby supporting the idea of variability amongst CO chondrites. Elfers *et al.*[11] also report μ^{96}Zr data for ordinary chondrites, which is in agreement with our data as well as Render *et al.*[10]. Schönbachler *et al.*[14], Render *et al.*[10] and Elfers *et al.*[11] report step leaching experiments for Murchison, which potentially allows comparison with our data. The 1b step of Schönbachler *et al.*[14] and the L1 step of Render *et al.*[10] uses the same protocol as our L3 step, making the data directly comparable. The μ^{91}Zr, μ^{92}Zr and μ^{96}Zr values of the L1 step of Render *et al.*[10] are 296±6, 124±6, 4998±18, respectively, which is identical to the values of 291.6±4.5, 122.1±3.1, 4977±12 we report for our L3 step. Although of lower precision, the $\mu\ ^{1}$Zr, μ^{92}Zr and μ^{96}Zr values of step 1b of Schönbachler *et al.*[14] are 260±60, 100±40 and 4860±140, respectively, also identical to our values. The step-leaching procedure utilized by Elfers *et al.*[11] is too different to allow for meaningful comparison. Finally, we note that Akram *et al.*[13] reports μ^{96}Zr data for refractory inclusions. Similarly to our data, they report ^{96}Zr excesses although their data are not as anomalous are we report, perhaps reflecting heterogeneity in the reservoir of refractory inclusions.

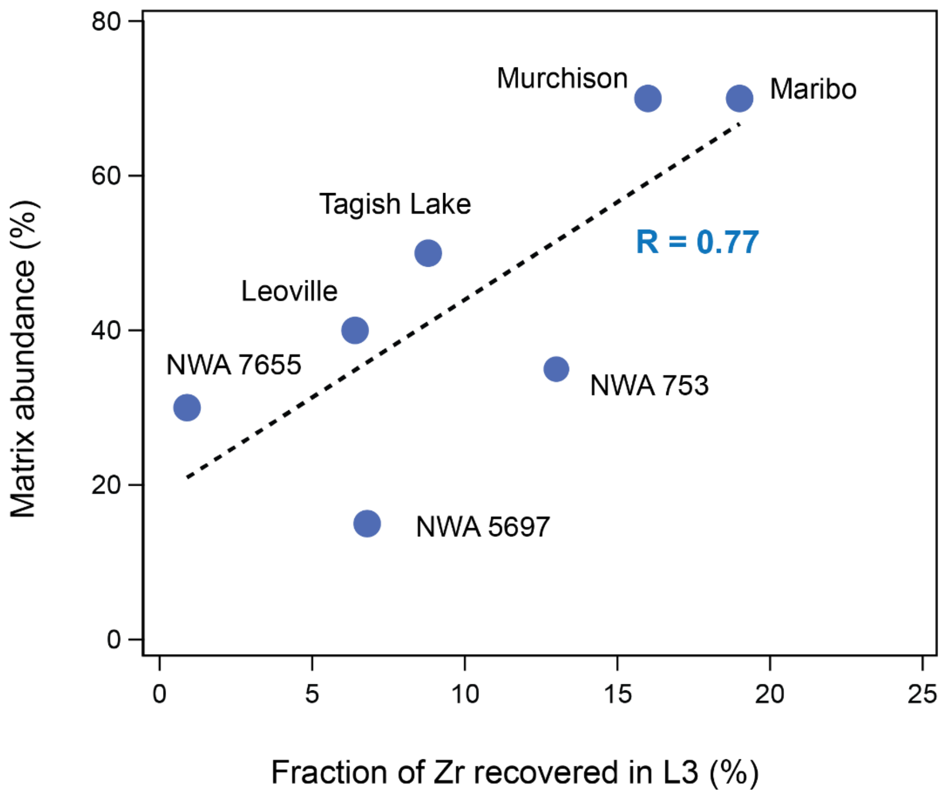

Fig. S1. Amount of Zr recovered in L3 fraction relative to the total Zr budget plotted against the estimated matrix abundances of the various meteorites. Matrix abundances are from [15,16] and the Zr data can be extracted from Data S1.

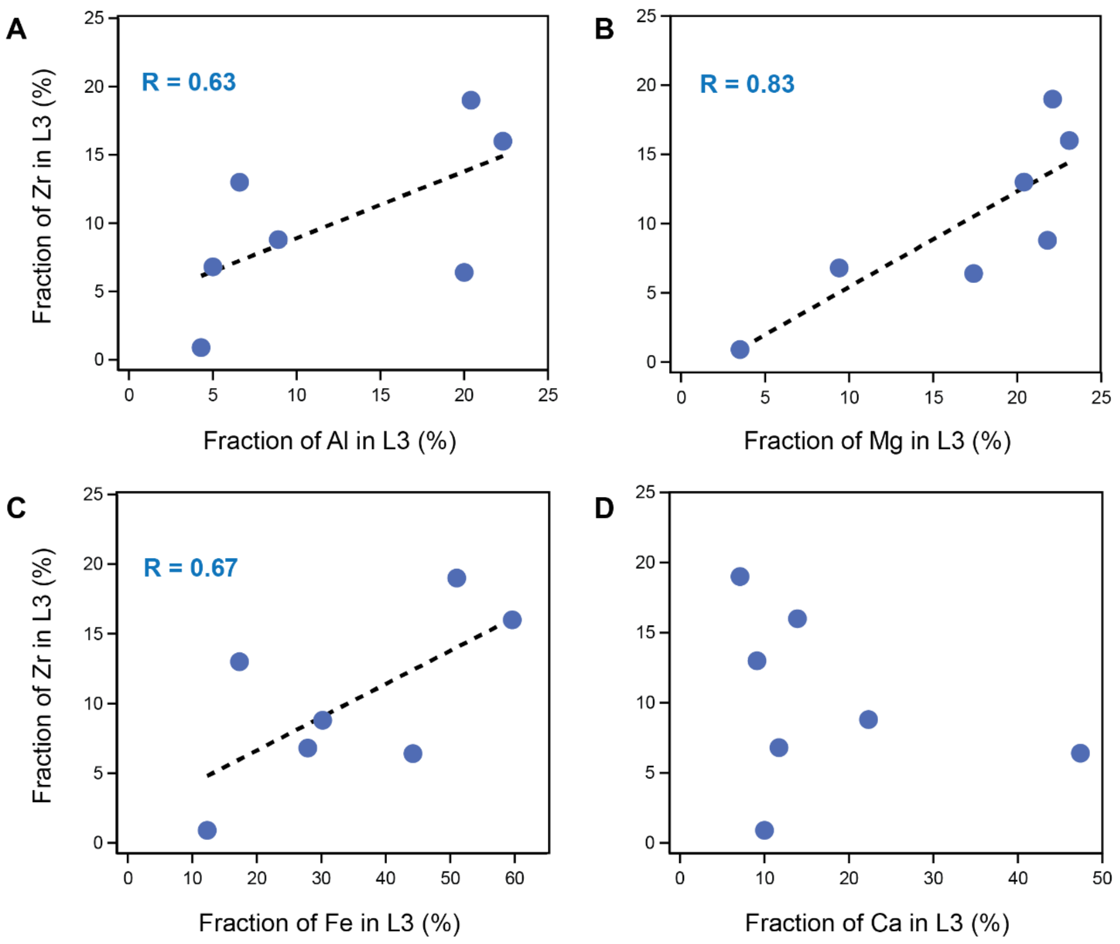

Fig. S2. Amount of Zr recovered in L3 fraction relative to the total Zr budget plotted against the Al (A), Fe (B), Mg (C) and Ca (D) in the same fraction relative to the total budget of each element. Data in these figures can be extracted from Data S1.

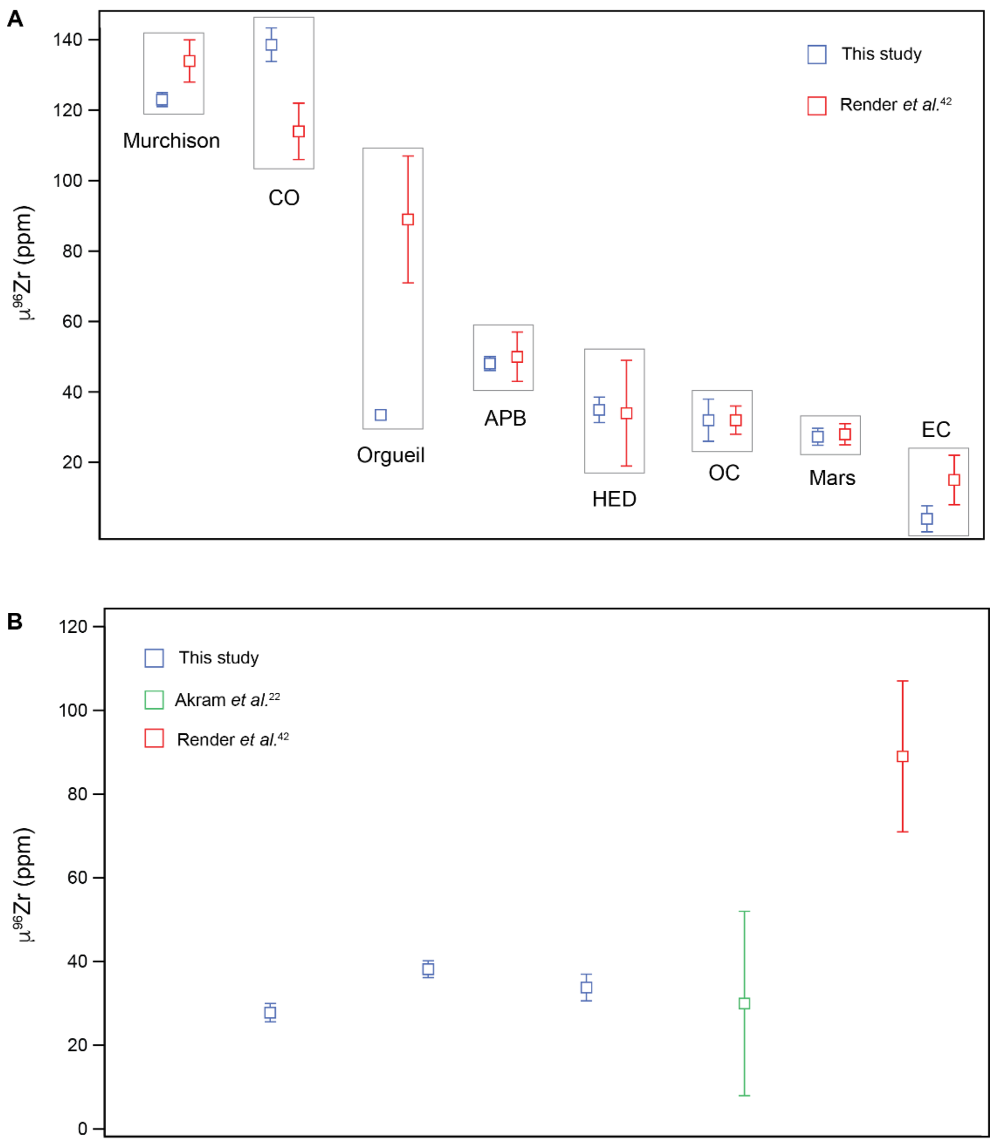

Fig. S3. Comparison of the μ^{96}Zr data reported in this study with recent literature data. In A, we compare our data with that of Render *et al.* [10] for the sample meteorites and/or meteorites classes. Uncertainties reflect quoted internal errors for single meteorites or weighted means and corresponding 95% confidence interval for meteorite classes. In B, we compare our Orgueil data with that of Akram *et al.* [9] and Render *et al.* [10]. APB, angrite parent body, HED, howardite-eucrite-diogenite, OC, ordinary chondrite, EC, enstatite chondrite.

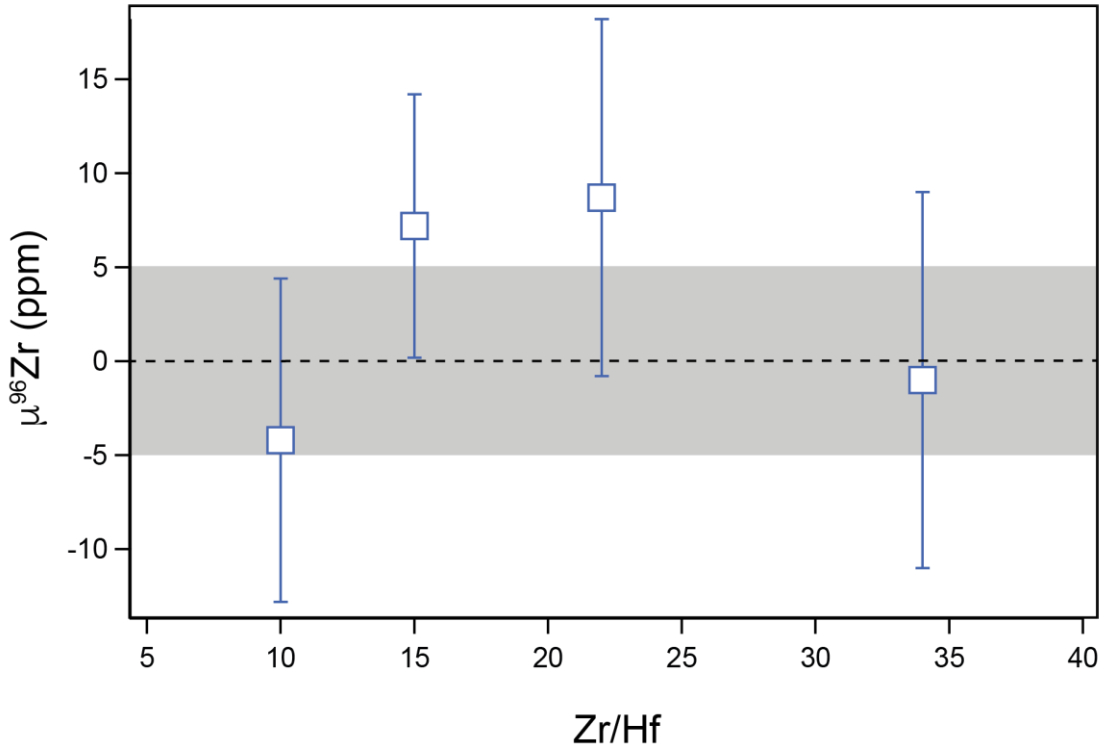

Fig. S4. Evaluation of doubly charged Hf interference on Zr isotope masses used for $\mu^{96}Zr$ determination. To test whether doubly charged Hf species (e.g., $^{192}Hf^{2+}$) introduce measurable isobaric interference on Zr isotope masses used in $\mu^{96}Zr$ determination, we conducted doping experiments using a Zr standard ICPMS solution spiked with increasing concentrations of Hf. The resulting solutions span Zr/Hf atomic ratios from 34 down to 10—up to a factor of ~3 lower than the least favorable Zr/Hf ratio measured in any of the meteorite samples analyzed in this study. The doped solutions were run against the pure Zr standard solution. All solutions were processed identically and measured using the same MC-ICPMS protocol applied to the sample set, and each doped solution was analyzed 5 times. The $\mu^{96}Zr$ values of the doped solutions are indistinguishable from the undoped reference solution within analytical uncertainty, demonstrating that potential interferences from Hf^{2+} species do not affect the accuracy of $\mu^{96}Zr$ measurements at the <10 ppm precision level achieved in this study.

Table S1. Zirconium isotope data for leaching experiments, whole rock meteorite samples and inclusions as well as terrestrial rock standards. N, number of sample analyses, O, ordinary, R, Rumuruti, UNC, ungrouped carbonaceous, HED, howardite-eucrite-diogenite.

**Sample**	**Type**	**μ^{91}Zr**	**2SE**	**μ^{92}Zr**	**2SE**	**μ^{96}Zr**	**2SE**	**N**
Leaching experiments								
NWA 5697 L3	O chondrite	88.4	10.4	37.5	7.0	1455.0	17	1
NWA 5697 Residue	O chondrite	-0.1	2.0	-2.7	3.4	-38.8	7.1	10
NWA 753 L3	R chondrite	-5.5	13.0	9.5	10.0	63.0	23	1
NWA 753 Residue	R chondrite	4.6	4.0	-2.2	3.5	26.5	8.1	6
Leoville L3	CV3 chondrite	202.7	8.7	80.6	3.8	3192.0	14	3
Leoville Residue	CV3 chondrite	-3.3	2.2	2.9	1.3	23.5	6.2	10
Tagish Lake L3	UNC chondrite	307.0	13.0	124.4	9.5	4942.0	24	1
Tagish Lake Residue	UNC chondrite	-4.3	7.9	-5.8	5.0	-117.0	12	6
Murchison L3	CM chondrite	291.6	4.5	122.1	3.1	4977.0	12	5
Muchison Residue	CM chondrite	-51.4	8.3	-23.6	4.9	-886.0	13	7
Maribo L3	CM chondrite	300.9	2.3	114.0	0.4	4859.3	3.4	3
Maribo Residue	CM chondrite	-45.9	3.4	-18.5	2.1	-696.0	14	10
NWA7655 L3	CR chondrite	152.9	2.4	61	15	2540	19	2
NWA7655 Residue	CR chondrite	-4.7	2.1	-2.1	2.9	87	16	5
Bulk chondrites								
SAH 99544	CO chondrite	3.5	1.3	2.1	0.7	138.6	4.8	8
Tagish Lake	UNC chondrite	5.6	1.9	2.7	2.0	154.2	7.2	4
Tarda	UNC chondrite	4.2	1.6	-0.2	2.5	123.4	6.2	10
Murchison (1a)	CM chondrite	1.4	2.1	2.9	1.8	120.0	6.0	10
Murchison (1b)	CM chondrite	0.6	1.7	1.6	1.5	125.5	5.3	10
Murchison (2)	CM chondrite	4.9	3.0	4.1	2.6	123.6	3.5	10
Murchison (3)	CM chondrite	4.2	2.8	1.4	2.2	123.3	2.8	10
Orgueil (1)	CI chondrite	2.7	1.5	8.5	0.9	27.8	2.2	10
Orgueil (2)	CI chondrite	3.8	2.5	8.1	1.2	38.2	2	10
Orgueil (3)	CI chondrite	2.6	2.2	5.5	2.3	33.8	3.2	10
SAH 97159	EH3	3.0	1.5	1.8	2.2	3.0	4.0	10
Atlanta	EL6	-3.3	3.1	-1.7	1.5	11.3	10.8	6
Plainview	H5	-6.6	3.3	-2.3	3.8	28.8	8.9	6
Raglan	LL3	-1.3	3.8	0.5	3.8	37.5	11.8	6
Roosevelt	H3	2.8	2.5	-1.4	3.1	32.2	11.7	9

Table S1 (continued).

Sample	Type	μ^{91}Zr	2SE	μ^{92}Zr	2SE	μ^{96}Zr	2SE	N
Bulk achondrites								
Juvinas	HED	1.5	1.2	-1.1	1.0	34.9	3.6	10
SAH99555	Angrite	1.7	0.9	-3.1	0.9	48.1	2.0	9
Zagami (1)	Shergottite	-0.6	2.0	2.3	1.6	31.5	4.8	10
Zagami (2a)	Shergottite	-0.7	1.7	-1.0	2.0	27.5	4.1	10
Zagami (2b)	Shergottite	0.5	1.3	-2.1	1.0	28.5	3.3	10
NWA 2975	Shergottite	1.5	1.2	0.6	1.7	27.3	2.6	10
Nakhla	Nakhlite	2.5	1.2	-1.7	1.6	22.0	5.1	7
Refractory inclusions								
CAI E31	^{26}Al-rich	18.5	3.7	24.3	4.3	296.0	17.0	3
CAI B12	^{26}Al-rich	14.6	4.4	16.9	3.8	311.2	43.0	2
AOA B18-2	^{26}Al-rich	16.5	2.1	14.4	2.9	317.3	6.2	3
KT-1	^{26}Al-poor	-19.5	2.5	-48.8	2.2	-293.2	6.8	6
STP-1	^{26}Al-poor	-17.0	5.4	-2.9	4.7	-264.6	31.0	1
Terrestrial standards								
BIR-1 (1)	Basalt	-0.3	1.1	0.0	1.6	2.2	4.0	9
BIR-1 (2)	Basalt	2.1	2.2	2.3	2.0	-0.3	5.4	9
BIR-1 (3)	Basalt	-0.6	4.8	3.1	2.5	3.4	3.1	5
BHVO-2 (1)	Basalt	-1.2	1.1	-0.9	1.1	-0.4	4.5	10
BHVO-2 (2)	Basalt	1.4	1.1	1.1	1.4	0.7	1.9	10
BHVO-2 (3)	Basalt	2.3	1.3	2.3	1.6	-0.6	2.1	10
BHVO-2 (4)	Basalt	1.7	1.1	3.0	1.5	-5.3	5.5	10
BCR-2 (1)	Basalt	-0.4	1.4	-1.1	2.3	-5.4	5.1	10
BCR-2 (2)	Basalt	-0.3	1.3	-0.9	1.4	0.1	2.8	10
BCR-2 (3)	Basalt	-0.2	1.3	-1.3	1.1	-1.6	9.9	10

Supplementary References

1. Alexander, C. M. O. Quantitative models for the elemental and isotopic fractionations in chondrites: The carbonaceous chondrites. Geochimica et Cosmochimica Acta 254, 277–309 (2019).

2. Brearley, A. J. The Action of Water. in Meteorites and the Early Solar System II (eds Lauretta, D. S. & McSween Jr., H. Y.) 584–624 (University of Arizona Press, Tucson, 2006).

3. Marrocchi, Y., Bekaert, D. V. & Piani, L. Origin and abundance of water in carbonaceous asteroids. Earth and Planetary Science Letters 482, 23–32 (2018).

4. Lodders, K. Solar System Abundances and Condensation Temperatures of the Elements. ApJ 591, 1220 (2003).

5. Suttle, M. D., King, A. J., Schofield, P. F., Bates, H. & Russell, S. S. The aqueous alteration of CM chondrites, a review. Geochimica et Cosmochimica Acta 299, 219–256 (2021).

6. Truelove, J. K. & McKee, C. F. Evolution of Nonradiative Supernova Remnants. The Astrophysical Journal Supplement Series 120, 299–326 (1999).

7. Pannuti, T. G., Rho, J., Heinke, C. O. & Moffitt, W. P. The X-Ray Properties of Five Galactic Supernova Remnants Detected by the Spitzer GLIMPSE Survey. The Astronomical Journal 147, 55 (2014).

8. Burkhardt, C. et al. Terrestrial planet formation from lost inner solar system material. Science Advances 7, eabj7601 (2021).

9. Akram, W., Schönbächler, M., Bisterzo, S. & Gallino, R. Zirconium isotope evidence for the heterogeneous distribution of s-process materials in the solar system. Geochimica et Cosmochimica Acta 165, 484–500 (2015).

10. Render, J., Brennecka, G. A., Burkhardt, C. & Kleine, T. Solar System evolution and terrestrial planet accretion determined by Zr isotopic signatures of meteorites. Earth and Planetary Science Letters 595, 117748 (2022).

11. Elfers, B.-M., Sprung, P., Messling, N. & Münker, C. The combined Zr and Hf isotope inventory of bulk rock and sequentially leached chondrite samples. Geochimica et Cosmochimica Acta 270, 475–491 (2020).

12. Render, J. & Brennecka, G. A. Isotopic signatures as tools to reconstruct the primordial architecture of the Solar System. Earth and Planetary Science Letters 555, 116705 (2021).

13. Akram, W., Schönbächler, M., Sprung, P. & Vogel, N. Zirconium-Hafnium isotope evidence from meteorites for the decoupled synthesis of light and heavy neutron-rich nuclei. ApJ 777, 169 (2013).

14. Schönbächler, M. et al. Nucleosynthetic zirconium isotope anomalies in acid leachates of carbonaceous chondrites. Geochimica et Cosmochimica Acta 69, 5113–5122 (2005).

15. Zolensky, M. E. et al. Mineralogy of Tagish Lake: An ungrouped type 2 carbonaceous chondrite. Meteoritics & Planetary Science 37, 737–761 (2002).

16. Scott, E. R. D. Chondrites and the Protoplanetary Disk. Annual Review of Earth and Planetary Sciences 35, 577–620 (2007).
