# Supplementary material for: Interstellar ices as carriers of supernova material to the early solar system
Source: Nat Commun. 2025 Nov 27;16:10657. doi: 10.1038/s41467-025-65672-5 (PMC12660829; doi:10.1038/s41467-025-65672-5)
Supplement: Supplementary file 2 — Description of Additional Supplementary Files [file 41467_2025_65672_MOESM2_ESM.pdf]

Description of Additional Supplementary Files:

Supplementary Data 1: Major and selected trace element abundances of leach and residue fractions.
